# Community drug distributors for mass drug administration in neglected tropical disease programmes: systematic review and analysis of policy documents

**DOI:** 10.7189/jogh.09.020414

**Published:** 2019-12

**Authors:** Cara L Macfarlane, Laura Dean, Rachael Thomson, Paul Garner

**Affiliations:** 1 Centre for Evidence Synthesis in Global Health, Department of Clinical Sciences, Liverpool School of Tropical Medicine, Liverpool, UK; 2 Department of International Public Health, Liverpool School of Tropical Medicine, Liverpool, UK; 3 Department for Tropical Disease Biology, Liverpool School of Tropical Medicine, Liverpool, UK

## Abstract

**Background:**

Mass drug administration (MDA) programmes for neglected tropical diseases (NTDs) depend on voluntary community drug distributors (CDDs) to deliver drugs, and these volunteer schemes need regular training and supervision. NTD policy now includes integration of multiple disease programmes, but we are unsure if there is clarity in what is currently expected of CDDs and how they are managed. We therefore analysed World Health Organization (WHO) policy, strategy and implementation guidance, and select national NTD programme implementation plans.

**Methods:**

Included are a) WHO global and WHO-Regional Office for Africa guidelines, strategies, operational manuals and meeting reports published between January 2007 to February 2018 that included policy and plans for CDDs; and b) national NTD programme master plans for Cameroon, Ghana, Liberia and Nigeria. For both review components, we examined the CDD responsibilities through a framework developed iteratively against the documents and prepared a narrative synthesis.

**Results:**

Twenty WHO policy documents met the inclusion criteria. In the twelve global and eight regional documents, the CDD role was not explicitly or comprehensively defined. Three documents mentioned CDDs will distribute drugs; some mentioned health promotion, data handling and engagement in clinical care. Four WHO documents noted a need for CDD training or management, eight detailed some aspect of this, and one regional document provided a comprehensive overview. In the national plans, additional responsibilities included case management in two countries and transmission control in two countries. Every plan included training and supervision, but this was not always explicit, and details of the purpose and frequency varied. In all national plans, CDD motivation was identified as a challenge but not comprehensively addressed, although one document mentioned provision of bicycles.

**Conclusions:**

WHO and national policies and plans assume CDDs will implement NTD programmes. However, there is almost no clear delineation of responsibilities, nor is there up-to-date practical guidance to guide managers. This ambiguity, in relation to the lack of explicit policies or programmatic guidance, probably impairs the effectiveness of NTD programmes.

## This project

We were part of development teams to supporting sustainable scaling up of mass drug administration (MDA) campaigns in Cameroon, Liberia, Ghana and Nigeria (http://countdown.lstmed.ac.uk/), and were asked to examine the evidence around volunteer community drug distributors (CDDs) in neglected tropical disease (NTD) programmes.

In our initial appraisal, we identified clear benchmarks in relation to a World Health Organization (WHO) affiliated training document for CDDs published in 1998 [1] but found more recent policies relating to CDDs were not as well-defined. As this cadre appears to be central to global programmes distributing freely donated drugs [2], we wondered how clear are policies and procedures set by WHO or national programmes to assure their distribution by CDDs? What are the explicit responsibilities of CDDs in contemporary MDA programmes for NTDs in Africa?

We address these questions in this analysis, but we first provide a brief overview of the history of NTD policy from 1995 to present day. We then systematically sought and analysed the CDDs role as presented in a) global and regional policy documents for Africa; and b) in select national NTD programme master plans.

## Historical overview

The WHO currently recognises 20 communicable diseases prevalent in tropical and subtropical conditions worldwide [3], collectively called neglected tropical diseases or NTDs. NTDs are associated with poverty, and primarily impact populations in low-income and middle-income countries with limited access to under-resourced health services [4]. NTDs are a diverse group of infections and conditions that adversely impact mental health [5], and cause considerable morbidity [6], social stigma [7] and death [8]. The African Region alone suffers around 40% of the global burden of NTDs [9].

Five NTDs – onchocerciasis, lymphatic filariasis, soil-transmitted helminthiasis (STHs), schistosomiasis and trachoma – can be managed through preventive chemotherapy (PC) and are termed PC-NTDs. When given annually or semi-annually to the entire population at risk (MDA) or specific at-risk groups (targeted treatment), PC can treat individuals for the infection, prevent new morbidity developing over time and reduce or interrupt disease transmission [10]. Of the 47 countries in the WHO African Region, 44 are endemic for one or more NTDs that are amenable to PC, and 17 countries are co-endemic for five of these diseases [2].

Control programmes require repeated rounds of drug delivery over several years to endemic areas, with high therapeutic and geographic coverage, to meet the public health targets for the PC-NTDs. Community-based delivery of PC is dependent on a cadre of health volunteers, termed community drug distributors or CDDs, to distribute treatment within their communities, particularly in hard to reach and remote areas [2].

## Origins

The community-directed treatment (ComDT) approach engaging volunteer CDDs for drug distribution was developed by the WHO and Special Programme for Research and Training in Tropical Diseases (TDR), and subsequently adopted by the African Programme for Onchocerciasis Control (APOC) in the late 1990s after a multi-country study showed that use of CDDs was a feasible and effective approach to deliver ivermectin for onchocerciasis [11]. This strategy became known as community-directed treatment with ivermectin (CDTI). CDDs were related but seen as separate to community health workers (CHWs), who often had a broader role in communities with health promotion and advice and could be paid for their services.

The “Practical Guide for Trainers of Community-Directed Distributors” was published by APOC in 1998, and CDDs were described as “villagers who have been selected by their communities to carry out the task of distributing ivermectin to other members of their community” [1]. This document was extensive and included detailed guidance on how CDDs should be selected, how to conduct their training and tasks for the trainee CDD post-training. Responsibilities of CDDs included conducting a village census to determine the quantity of drugs required, keeping a drug inventory, keeping records and liaising with health workers, providing the correct drug dosage, excluding people ineligible for treatment (pregnant and breastfeeding women, sick people) and referring people experiencing adverse reactions to a health facility [1]. The community was responsible for incentivising CDDs, and sustainability of the programme stemmed from greater ownership and empowering communities to have control over their own health.

## Integration

Following the initial success of the CDTI strategy, which included 20 million people being treated annually and 67 000 CDDs trained by 2001 [12], the CDTI network was soon used to deliver additional community health services [12,13] and explored by other NTD programmes, including lymphatic filariasis [14] and schistosomiasis and STHs [15].

In 2003 and 2005, the WHO held international workshops that coined the collective term “neglected tropical diseases” and started the transition from vertical disease-centred approaches to an integrated community-centric approach [16,17]. The narrative changed to the promotion of integrated NTD programmes, where many of the PC-NTDs have overlapping geographical distributions and treatment regimens [10,18-21]. In 2006, the WHO launched a new drug strategy for integrated PC for helminthiasis [10], and the United States Agency for International Development (USAID) established the NTD Control Program (NTDCP) to test integration on a large scale and facilitate expansion of integrated treatment in select national programmes [22,23].

The first “Global Plan to combat Neglected Tropical Diseases (2008-2015)”, published by the WHO in 2007, then endorsed integrating NTD control programmes worldwide with co-implementation of PC as a ‘drug package’ [24]. The NTD Roadmap was later published to guide implementation of the policies and strategies outlined in the global NTD plan, and quickly followed by support from the London Declaration on NTDs [25].

In the WHO African Region, integrated PC is currently a priority intervention in the “Regional Strategy on NTDs in the WHO African Region (2014-2020)” and includes the co-implementation of community-directed interventions (CDI) [26]. Country-specific strategies for integrated NTD control and elimination are stipulated in the multi-year master plan for national NTD programmes, in accordance with WHO guidelines [27].

## Objective

To delineate the role of CDDs in contemporary MDA programmes for NTDs in the African Region, as defined in global and regional policy documents; and as outlined in a selection of national NTD programme master plans.

## METHODS

### Inclusion criteria

For the policy documents, we included documents from WHO global programmes and documents from the WHO Regional Office for Africa (WHO-AFRO). We sought WHO guidelines, strategies, operational manuals and meeting reports published between January 2007 to February 2018 that included policy and plans for CDDs in PC-NTD programmes. Reports of meetings were included if there were recommendations or action points relating to CDDs. We took 2007 as the starting point given the establishment of worldwide NTD policy by the WHO in this year, which included the first global partner's meeting on NTDs [28] and publication of the global NTD plan [24]. This set the international agenda for NTD prevention, control, elimination or eradication, and we would expect any resultant changes in the expectations of CDDs and their management to be outlined clearly in the policies and plans that followed.

Exclusion criteria consisted of documents described as “working drafts”, reports, documents that only referred to CDDs and related terms in the introductory sections or in a “situation analysis”, “update” or “progress report”, and documents that referred only to CDI or CDTI without clear reference to CDD involvement.

For the second objective, we sought the NTD programme master plans from Cameroon, Ghana, Liberia, and Nigeria. These countries were selected as they all receive financial and technical support from the COU**NTD**OWN consortium (a Department for International Development (DFID)-funded project). These countries use the standard NTD programme template and were selected to be part of the COU**NTD**OWN consortium as they are all in different phases of NTD control (https://countdown.lstmed.ac.uk/).

### Definition of a community drug distributor

The CDD cadre has various labels and expectations, and so our definition is: “a volunteer who is selected by the community to distribute drugs for diseases targeted by NTD programmes”. This includes volunteers that participate in MDA to all members of a community and volunteers involved in targeted treatment of specific at-risk groups in the community [10]. Within the context of this review, we use a slightly modified definition of the CHW described by Lewin *et al* [29] to include any health volunteer who:

- performs functions related to drug distribution to prevent select NTDs;
- is trained in some way in the context of the intervention; but
- has no formal professional or paraprofessional certificate or tertiary education degree.

This definition of the CDD cadre excludes teachers, frontline health workers and other individuals who may be involved in drug distribution in conjunction with their salaried employment.

### Policy documents

#### Search

We attempted to identify all relevant WHO policy documents published between January 2007 to February 2018 by searching https://www.who.int with “Global” as the regional site (English only), the WHO-AFRO Library and the WHO IRIS AFRO collections (no language restrictions) using the search terms and subjects listed in Table S1 in **Online Supplementary Document**. We also searched the APOC website and the Expanded Special Project for Elimination of Neglected Tropical Diseases (ESPEN) portal resources for publications. We noted any documents that are currently in preparation or documents we are aware of but unable to obtain a copy. We engaged with experts from the research, advocacy and policy sectors in the field of NTDs to ensure inclusivity of all relevant policy documents.

#### Selection

Records identified from the search strategy were combined and titles screened to remove duplicate and irrelevant records. Records that were clearly not policy documents, such as WHO Weekly Epidemiological Reports, brochures and newsletters, were excluded.

#### Data identification

Full-text copies of all potentially relevant policy documents were obtained and assessed by one author based on the inclusion criteria using NVivo 11 Software (QSR International, Melbourne, Australia). Text search was used to identify sought-after text, such as “distributor”, in each document, applying the “with stemmed words” setting to identify similar text (eg, distributors). The list of text terms is provided in Table S1 in the **Online Supplementary Document**. When it was unclear, a second author was consulted. Ineligible documents and the reasons for their exclusion were listed in a “characteristics of excluded documents” table.

#### Data extraction

Pre-tested data extraction forms were used to manage the data. We extracted information on the type of document, the publication date, the scope of the document (global or regional policy), the disease(s) covered and statements relevant to the drug distributor identified through the NVivo text search. For each document, the statements were organised into groups, including: 1. assertions about their role; 2. recommendations for their role; 3. reference to their training and management; and 4. other comments about CDDs.

#### Data analysis

We prepared a narrative synthesis of the role of CDDs as is documented in global and regional documents. We examined the documents and the data and then used this to inform the categories. Documents were assessed using four broad categories that were subdivided to reflect the description of the CDDs role, the strength of the policy, and the level of detail provided:

1. *Specifying aspects of the CDDs role.* The main options were drug distribution, data collection, health promotion, and a role in clinical care.
2. *Recommendations for the CDDs role*. Options included: none, an option with caveats, an option, and a formal recommendation.
3. *Strategies for training and managing CDDs.* Options included: no strategies, notes need, comment on aspects, and clearly and comprehensively outlined.
4. Other comments about CDDs.

We considered “specifying aspects of the CDDs role” as a directive statement about an activity CDDs are or will be involved in, while “other comments about CDDs” were more general statements that included other relevant information about CDDs but were not instructional.

### National programme master plans

**Figure 1:**
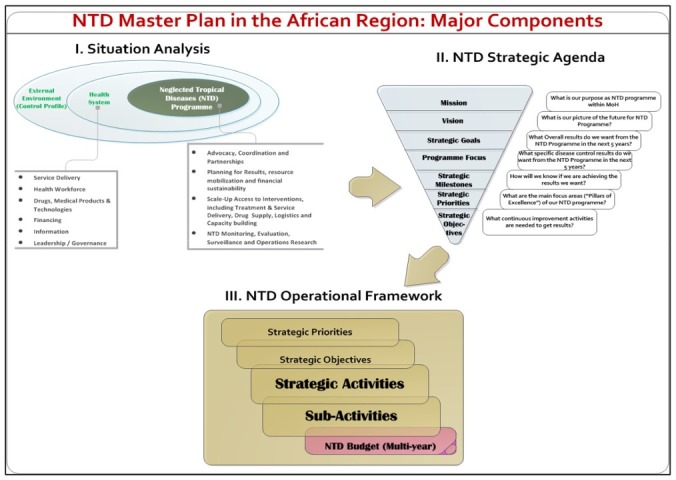
Major components of neglected tropical disease programme master plans for the African Region outlined by the World Health Organization [27].

The national NTD programme master plans follow the structure recommended in the WHO “Guide for preparing a master plan for national NTD programmes in the African region” (**Figure 1**) [27], and include the national objectives, goals and a 3-5 year strategy to guide programme planning and implementation. We obtained the most recent national NTD master plan from Cameroon, Liberia, Nigeria and Ghana through the COU**NTD**OWN consortium and the ESPEN portal resources.

#### Data extraction

Data extraction recorded the date of publication and period covered by the plan, the disease(s) covered and information relevant to the drug distributor, including the diseases requiring drug delivery by CDDs, any other activities CDDs are implicated in, details of training and supervision, and whether incentives are included. Prior to using text search to extract relevant data, we scoped the master plan glossaries and introductory “situation analysis” sections to identify any other country-specific terms for CDDs. We then conducted the same text search as was used for the global and regional documents, with the addition of any country-specific terms for CDDs and the terms supervis* and train*.

#### Data analysis

A narrative synthesis was prepared to document the intended role of CDDs as it is outlined in the NTD programme plans of Cameroon, Ghana, Liberia and Nigeria. Statements in the plans regarding CDDs were analysed by compiling information relating to:

1. *Anticipated role of CDDs*, plus any additional activities of volunteers mentioned in the “situation analysis”.
2. *Strategies for training and management*.
3. *Motivation and incentives* (motivation is acknowledged, and incentives included).

## RESULTS

### Policy documents

**Figure 2:**
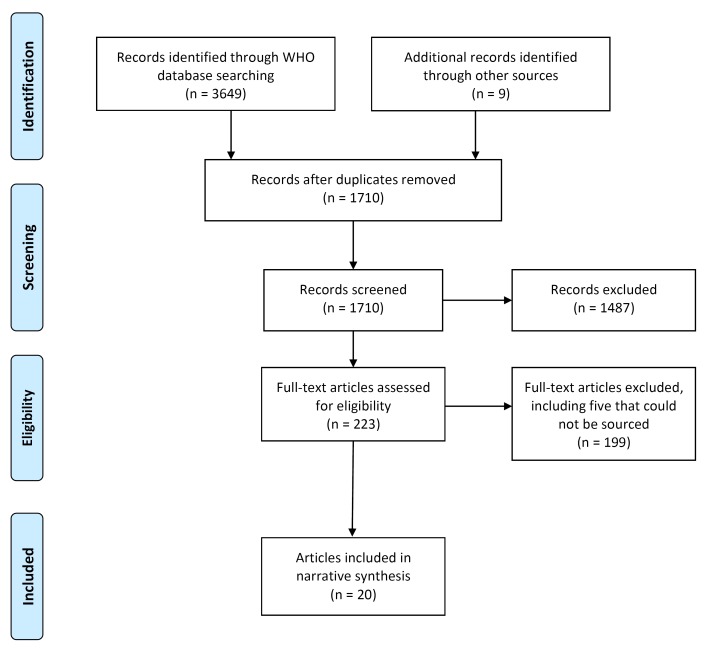
PRISMA flow diagram.

Twenty documents in 24 records met the inclusion criteria (**Figure 2**). One document, the regional strategy on NTDs in the WHO African Region [26], had three related records [26,30-32] but there was no difference in relation to content on CDDs, so we report this as one document [26]. Another document [33] had one related record [34], which was a shorter overview of a curriculum, and we report on the more comprehensive document [33]. We excluded 199 full-text documents that did not meet the inclusion criteria, including five documents that we were unable to source an electric copy of [35-39], and two documents that were “working drafts” and subject to further revision [40,41]. We included one “working draft” as it was recommended that the tool be made available to national programmes for immediate use [42].

#### Global

Of the 20 documents relevant to CDDs, 12 were global in scope. Six documents were about lymphatic filariasis (two described as “strategy”, and the other four variously described as “aide-memoire”, meeting report, handbook and manual; **Table 1**, column 1). The remaining six documents were about the PC-NTDS (described as guide, manual, practical advice, tool and training course (2)). Supportive evidence is provided in Table S2 in **Online Supplementary Document****.**

**Table 1:** Global documents

Column	1	2	3	4	5	6
**Year [ref]**	Type of document*	**Disease(s) covered**	**Specifying aspects of the CDDs role?**	**Recommendations for the CDDs role?**	**Strategies for training and managing CDDs?**	**Other comments about CDDs^†^**
2010 [43]	“Strategic plan”	LF	No	None	No	Included
2012 [44]	“Provisional strategy”	LF^‡^	No	An option with caveats	Notes need	None
2013 [45]	“Aide-memoire”	LF	No	None	Notes need	None
2016 [46]	“Meeting report”	LF	No	None	Comment on aspects	None
2016b [47]	“Guide”	PC-NTDs	No	None	Comment on aspects	Included
2013b [48]	“Handbook”	LF	No	An option	Notes need	None
2010 [49]	“Manual”	PC-NTDs	Data collection	None	Comment on aspects	Included
2011 [50]	“Manual”	LF	Data collection	None	Comment on aspects	Included
2011 [51]	“Practical advice”	PC-NTDs	Clinical care	None	No	Included
2016c [42]	“Tool”	PC-NTDs	No	None	Comment on aspects	Included
2015 [52]	“Training course”	PC-NTDs^§^	Drug distribution; data collection; health promotion; clinical care	None	Comment on aspects	Included
2015b [53]	“Training course”	PC-NTDs	Drug distribution; data collection; health promotion; clinical care	None	Comment on aspects	Included

CDD – community drug distributor, LF – lymphatic filariasis, PC-NTDs – preventive chemotherapy neglected tropical diseases, ref – reference

*Type of document is the words in the title that best classify the document.

†Comments about CDDs provided in the document’s “background/introduction”, or in a “progress report”, an “update” or a “current situation”, were not included as other comments about CDDs in policy.

‡Loiasis and malaria are also included in document.

§Also mentions foodborne trematodiases.

**Specifying aspects of the CDDs role:** Of the 12 documents, seven made no reference to the responsibilities of CDDs in MDA (**Table 1**, column 3). Two training documents specified that CDDs have a role in drug delivery, stating that “CDDs play a critical role in distribution” and are responsible for leading “absentee and post-MDA follow-up”. CDDs are also involved in distributing information, education and communication (IEC) materials and “other service delivery (MMDP)” (morbidity management and disability prevention). Some documents indicated that CDDs should carry out other tasks, including:

- collecting, recording and reporting information (four documents), such as completing PC tally sheet forms or registers, leading the community census and “monitoring inventory of logistics”.
- health promotion (two documents), including leading community meetings, sensitisation and mobilisation, conducting IEC activities before and while distributing interventions, and lymphoedema/hydrocele case finding by going door-to-door to “disseminate the message”.
- clinical management role (three documents), including assisting with adverse events (AEs) and referring people to a health facility, and record and report AEs. Actions for CDDs to manage AEs were provided, with a list of common AEs or symptoms, their frequency and treatment. There was an emphasis on drug safety (CDDs understanding treatment exclusion criteria, awareness of issues relating to treating children) and CDDs performing adequately (“CDDs must give the right information”, “CDD… and child should be relax, calm, friendly”).

None made clear exactly what CDDs should do, or how they should do it.

**Recommendations for the CDDs role:** There were no recommendations for CDDs. Two documents for lymphatic filariasis mentioned CDDs may be of use to malaria programmes by encouraging net use.

**Strategies for training and management:** No document provided a comprehensive training and management strategy for CDDs. Two documents did not mention the topic, and three commented on the need for training and supervision but gave no further information. In the remaining seven, two training course documents provided some information of practical help for training. For example, “CDDs are community members who will be selected by the communities to undertake the task of drug distribution”, the number of CDDs per hamlet to train (two), the gender ratio of CDD teams (“as much as possible choose a team of two people, a man and a woman”) and length of training period (between two days). A list of what to include in CDD training was provided: “Diseases: Very brief description of diseases targeted, MDA strategy; Drugs: What drugs to use, Dosage, including use of dose poles; How to administer, Exclusion criteria, AEs/SAEs identification, management and referral; Register/Tally Sheets”.

For CDD management, a training course included “Regular supervision and monitoring in the community”, and the responsibilities of the different personnel overseeing CDDs was detailed. Some documents highlighted a need to inform community volunteers of the results of their work and discuss problems, including “drug administration, remuneration, compliance, IEC, form filling, use of remaining drugs…”. Four documents included practical guidance for monitoring, with corrective actions to take with CDDs when interpreting and following up reported and surveyed coverage results, and in the event of Transmission Assessment Survey1 (TAS1) failure.

**Other comments about CDDs:** Scattered through the documents were statements that indirectly referred to the CDDs role and how they should be managed. This included benefits and challenges of integration for CDDs, such as “improved supervision of community drug distributors (CDDs)… with fewer resources” and “increasing demands are being placed on volunteers as other programmes recognize their potential”. One document noted that “…for volunteers to deliver chemotherapy for a variety of NTDs and provide other health services, it will be necessary for health systems to create clear job descriptions, standardize incentives across programmes, and coordinate efforts to build capacity”.

Other statements mostly centred around CDD performance, such as data quality concerns, operational errors and CDD attitude, and preparation for MDA, which consists of selection of CDDs (two people, a man and a woman, selected by the community or by district level personnel), mode of drug distribution (eg, house-to-house, from a central point, areas where people congregate or determined by the District NTD coordinator) and the MDA commodities required.

#### Regional

We identified eight regional documents. Five documents were about onchocerciasis (described as meeting reports (4) and handbook; **Table 2**, column 1), and one document was about onchocerciasis and lymphatic filariasis (described as “strategic plan of action”). The remaining two documents were about NTDs (described as “strategy” and meeting report). Supportive evidence is provided in Table S3 in the **Online Supplementary Document**.

**Table 2:** Regional documents

Column	1	2	3	4	5	6
**Year [ref]**	Type of document*	**Disease(s) covered**	**Specifying aspects of the CDDs role?**	**Recommendations for the CDDs role?**	**Strategies for training and managing CDDs?**	**Other comments about CDDs^†^**
2014 [26]	“Strategy”	NTDs	No	None	No	Included
2013 [54]	“Strategic plan of action”	LF and onchocerciasis	No	None	Comment on aspects	Included
2007 [55]	“Meeting report”	Onchocerciasis	No	Formal recommendation	No	None
2008 [56]	“Meeting report”	Onchocerciasis	No	None	No	Included
2009 [57]	“Meeting report”	Onchocerciasis	No	None	No	Included
2012 [58]	“Meeting report”	Onchocerciasis	No	None	Notes need	Included
2017 [59]	“Meeting report”	NTDs^‡^	No	An option	No	None
2012b [33]	“Handbook”	Onchocerciasis^§^	Drug distribution; data collection; health promotion	An option	Clearly and comprehensively outlined	Included

CDD – community drug distributor, LF – lymphatic filariasis, NTD – neglected tropical diseases, ref – reference

*Type of document is the words in the title that best classify the document.

†Comments about CDDs provided in the document “background/introduction”, or in a “progress report”, an “update” or a “current situation”, were not included as other comments about CDDs in policy.

‡Addresses preventive chemotherapy NTDs and case management NTDs.

§Also mentions other diseases that may use community-directed interventions, such as lymphatic filariasis, schistosomiasis, trachoma and malaria (bed nets).

**Specifying aspects of the CDDs role:** Of the eight documents, seven made no reference to the role of CDDs (**Table 2**, column 3). One document, a trainers’ handbook for a curriculum and training module on the CDI strategy for medical and nursing students, stated “Implementation of the intervention by community implementers” consists of “delivery of the interventions”. Specific tasks that CDDs are responsible for performing included:

- “attend training and other educational programmes in respect of the intervention programme;
- provide information, education and communication to the community;
- conduct census and estimate the population that will need the service;
- collect or ensure that intervention commodities are available in the community;
- keep record of how the intervention was delivered;
- submit a record of intervention to the supply collection point;
- provide feedback to the community.”

The document outlines the community’s role in planning and decision-making for CDI, although given that the decisions about the drugs and the diseases targeted are already made, it is not clear what these decisions are really. There is almost no guidance for appropriate CDD workload and the number of interventions or other services volunteers can be responsible for.

**Recommendations for the CDDs role:** One of the eight documents made a recommendation about CDDs. The Joint Action Forum (JAF) for onchocerciasis control “strongly endorsed” a change in the ratio of CDDs to people treated, from one CDD for up to 250 people to one CDD for up to 100 people. One document provided an option for CDDs to carry out sensitisation on skin NTDs during MDA and one document listed several potential interventions for volunteer distribution, including maintain and distribute bed net supplies, and case management of malaria, pneumonia and diarrhoea.

**Strategies for training and management:** Five documents did not mention the topic, and one endorsed the need for training more women CDDs but no further information. One document provided a few vague options for managing CDDs dwindling motivation to work on a voluntary basis over an extended period.

One document outlined a comprehensive curriculum on the CDI strategy for students in medical and nursing schools. The guidance in this trainers’ handbook included units on “setting up the CDI strategy”, “supervision” and “monitoring and evaluation”. Setting up the CDI strategy in a community was described in detail and included use of the APOC video for training CDDs. Instructions for conducting CDD training were not included but use of the original APOC training manual for CDDs was recommended, with audio visuals by NTD support initiatives where the training requires multiple interventions, such as distributing bed nets and drugs for other NTDs. Two to three days should also be allocated for training CDDs.

For management, the CDI trainers’ handbook included the frequency of supervision for CDDs (twice monthly), the supervisor role and tools, and examples of forms and checklists for supervision and monitoring (such as “Integrated supervision checklist for PHC programmes” and “Supervision checklist for CDTI”). Examples of problems that can be encountered during supervision and possible solutions/actions were provided, as well as examples of CDD supervision tasks.

**Other comments about CDDs:** Statements concerning CDDs were included in six of the eight documents. The trainers’ handbook for CDI contained general statements throughout, including the community’s role in incentivising CDDs, CDD selection (community informed of selection criteria and then community selects implementers) and modes of drug distribution (door-to-door, community visits volunteer’s home, or at a central place). Two other documents included statements regarding incentives for CDDs, and one document acknowledged CDDs use in case finding for “eye care”. The AFRO-regional strategy stated that “Cross-cutting interventions or activities… should be harmonized and streamlined…”. Listed among the interventions and activities was “involvement of community volunteers or medicine distributors”. For supporting morbidity management, one document specified that “involvement and collaboration of patients and their families, community volunteers and community health workers is essential”.

### National programme master plans

We obtained the current NTD master plan for Cameroon (2016-2020), Ghana (2016-2020), Liberia (2016-2020) and Nigeria (2015-2020). The Cameroon master plan is currently a draft and available in French only. This plan was translated to English, and we acknowledge that the plan may be less formulated in its’ current format as a draft document. The plans range from 61 to 148 pages in length and consist of the main WHO recommended sections [27], including: situational analysis, NTD strategic agenda and operational framework, as well as budget justification and estimates (**Figure 1**). Our analysis is summarised in **Table 3**.

**Table 3:** The role of community drug distributors in national neglected tropical diseases programme master plans for Cameroon, Ghana, Liberia and Nigeria*

	National plan		NTD programme master plan			
**Country**		**Cameroon**	**Ghana**	**Liberia**	**Nigeria**
**Period**		**2016-2020**	**2016-2020**	**2016-2020**	**2015-2020**
**Anticipated role of CDDs**	**NTD programme**					**Disease**
	Drug distribution	Onchocerciasis	MDA	MDA	MDA	MDA
		LF	MDA	MDA	MDA	MDA
		Schistosomiasis	Targeted	Targeted	Targeted	Targeted
		STHs	Targeted	Not required	Targeted	Targeted
		Trachoma	MDA	Not required	Not required	MDA
		Other NTDs	Not required	Not required	Unclear*	Not required
	Case management and chronic care		Not included	Yes	Yes	Not included
	Transmission control (eg, bed net delivery)		Not included	Not included	Yes	Yes
	Other activities documented (eg, vitamin A distribution)		Yes	Not included	Yes	Yes
**Training**	Required by plan?		Required	Required	Required	Required
	Who will train?		Not specified	“Personnel”	Not specified†	Not specified‡
	Frequency of training		Annually	Q2 and Q4	Q1, Q2, Q3, Q4, p.a.	Annually§
**Supervision**	Required by plan?		Required‖	Required	Required	Required
	Who will supervise?		Not specified	“Personnel”	Not specified†	Not specified
	Frequency of supervision		Annually‖	Q2, Q3, Q4. Monthly sub-district review meeting (CDDs)¶	Not specified. Monthly meeting with health facility OIC (CHVs)	Not specified
**Incentives**	Included in plan?		Unclear**	Not included	Bicycles	Usable IEC materials

CDD – community drug distributor, CHV – community health volunteer, IEC – information, education and communication, LF – lymphatic filariasis, MDA – mass drug administration, NTD – neglected tropical diseases, OIC – Officer in Charge, p.a. – per annum, STH – soil-transmitted helminths

*We refer to “MDA” when mass drug administration is indicated, and “targeted” when treatment appears to be indicated for specific groups. We anticipated the distributors involvement when the drug delivery strategy included terms such as “community-based”, “community directed intervention (CDI)”, and “community directed treatment with ivermectin (CDTI)”. The Liberia plan indicates community MDA for yaws but it is not clear whether this requires community volunteers or “staff”.

†The Liberia plan did not identify the trainer and supervisor of CDDs, although the “situation analysis” indicates this requires “health facility staff”. For case management NTDs, cascade training will be used.

‡Training of CDDs for MDA is not described in the Nigeria plan. Training community implementers on the use of “LLINs, IRS and personal hygiene” requires training of health workers.

§The Nigeria plan includes annual training of “community NTD implementers on the use of data collection reporting tools”, but overall training for CDDs is not indicated.

‖The Cameroon plan specifies “supervision of community-based actions”.

¶The Ghana plan states “conduct monitoring and supervision at all levels” for MDA. Sub-district monthly review meetings require “transport for CDDs”.

**“Motivate community actors” annually is in Cameroon’s plan but no further details.

**Anticipated role of CDDs:** Each plan includes a table summarising the disease-specific goals and objectives, which generally indicates the main strategies/interventions, delivery channels and target population for each targeted disease. Community-based MDA is the strategy for onchocerciasis and lymphatic filariasis in all four plans, and for trachoma in Nigeria and Cameroon where the disease is endemic. All countries are endemic for schistosomiasis and STHs, and the strategy for schistosomiasis in the four plans and for STHs in three plans (excluding Ghana) consists of community-based targeted treatment to specific at-risk groups. Use of CDTI/CDI is indicated as the “delivery channel” in some of the strategies, but only the Liberia plan specifically mentions “community health volunteers” for STHs. Three of the plans (excluding Cameroon) provide a table detailing the packages of MDA, with “training community volunteers” as an overall requirement, implying their involvement in drug delivery. MDA is also included for yaws in two plans (Ghana and Liberia), but this requires health workers in Ghana and it is unclear whether community-wide treatment requires “staff” or volunteers in Liberia.

For case management and chronic care, two plans (Ghana and Liberia) include NTD volunteers. In Ghana, volunteers will conduct “Case search and management”, and in Liberia, volunteers are implicated in routine active case detection and referral for ‘case management’ NTDs. For transmission control, two plans (Liberia and Nigeria) include volunteers. In Liberia, a community health volunteer (CHV) in each community will distribute insecticide treated nets (ITNs), and in Nigeria, community volunteers will be trained to use “LLINs, IRS and personal hygiene” (long lasting insecticidal nets, indoor residual spraying). The Liberia plan also includes health promotion, such as conducting meetings and providing kits and training to “affected individuals” to enable “home based self-care for CM-NTDs” (case-management NTDs).

Two plans (Cameroon and Liberia) acknowledge other activities NTD volunteers have been involved with, including: delivering bed nets, vitamin A and immunisations, “family planning interventions ORS and zinc”, identifying scrotal swelling and lymphedema cases, detection and referral of suspicious leprosy cases, social mobilisation, contact tracing, health education and “member of burial teams…during the EVD outbreak”. The Nigeria plan also states that CDTI provided entry points for other community-based interventions.

**Strategies for training and management:** All the country master plans include aspects of training and supervision, but details on the frequency and individuals responsible for training and managing CDDs are not always described.

For training, the Cameroon plan specifies annual training of “community agents” and “community actors in social mobilization”, and community relay training in data management. For Ghana, “training of… community based volunteers will be done… jointly for all the diseases”. CDDs will be trained to conduct MDA by “personnel” twice a year. The Liberia plan includes training of community volunteers “on integrated NTD activities” quarterly over 2017-2020, and training of a CHV in the third quarter annually to distribute bed nets. For case management, “… community volunteers (especially CDDs) shall be trained in the suspicion cases during CDI activities, and referral of suspected cases to the health facilities...”, with integrated in-service cascade training of CHVs. For Nigeria, a priority is “comprehensive capacity building of NTD staff and volunteers at all levels on integrated NTDs”. The plan includes annual training of community NTD implementers to use “data collection reporting tools”, and on the use of “LLINs, IRS and personal hygiene” (requiring training of health workers to conduct training).

For supervision, the Cameroon plan includes “supervision of community-based actions” annually. In the Ghana plan, “personnel” and district staff are needed to monitor CDD training over 2013–2017. For MDA, “personnel” are required to “conduct monitoring and supervision at all levels” in periods “Q2, Q3, and Q4”. Sub-district monthly reviews over 2013-2017 will also require “transport for CDDs”. Supervision is listed as a requirement in the Liberia and Nigeria plans, and Liberia also plans to “integrate NTDs into monthly meeting of CHVs/CHA with health facility OIC to report on conditions within the community”. However, no further details on CDD management are provided.

**Motivation and incentives:** As part of the situation analysis, all plans include a strengths, weaknesses, opportunities and threats (SWOT) analysis for the NTD programme. The plans all include the presence of “mobilizable”, “committed” or “trained” CDDs in communities as a strength or opportunity, but also note challenges. The Cameroon and Ghana plans indicate lack of motivation as an issue, as well as the demand for incentives and ageing of the distributors. The Ghana and Liberia plans recognise that volunteers can be demotivated by other non-governmental organisation (NGO) activities and competing health activities and incentives between programmes. The Liberia plan states that “… lack of funding and incentives are an ongoing challenge for CDDs”, but CDDs could be involved in income generating activities through collaboration with development NGOs and Agriculture Ministry. For the Nigeria NTD programme, high attrition, low commitment and inadequate numbers of trained community implementers are challenges.

For proposed activities, the Cameroon plan states “encourage communities to document motivation mechanisms of community workers”, with development and dissemination of “motivation reporting tools for community workers”. “Motivate community actors” annually is also part of the plan, but no details are provided. Incentives for CDDs in Liberia include “procure 3000 bicycles for CDDs” in the second quarter of 2017. In Nigeria, a priority is the “provision of incentives in form of usable IEC materials”. The Ghana plan does not include incentives for CDDs.

## DISCUSSION

The role of CDDs has changed over the years since APOC first started using the CDTI implementation model. With the intensified drive to meet current global health targets for NTDs controlled by drugs, delivery of PC by community volunteers appears critical for programme success but could potentially be a weak link [60-62]. This review consolidated the evidence from more recent WHO global and WHO-AFRO regional documents and select national NTD programme master plans in Africa. We found that the policies and plans assume CDDs will implement NTD programmes, but there was little delineation of responsibilities or up-to-date practical guidance. This may signal an absence of explicit policy and programme management, and this ambiguity probably impairs the effectiveness of these programmes.

Only three documents explicitly stated that CDDs will distribute drugs. Other tasks associated with drug distribution, such as health promotion, data collection and reporting, and clinical care, were also specified in few documents. These activities are fairly consistent with what was expected of CDDs under single-disease control guidance from APOC [1]. Additional activities identified in the more recent policy documents included “MMDP delivery”, although what this constitutes was unclear. Some documents provided potential uses for CDDs, such as in delivery of bed nets and sensitisation for skin NTDs, and only one document from 2007 made a recommendation relating to CDDs [55]. Similarly, the role of CDDs in the national multi-year master plans was not explicit. For NTDs requiring drug distribution, the plans refer to community-based, use of CDTI/CDI structures, or MDA, with “volunteer training” listed as a requirement alongside these strategies. Half of the country master plans also involve volunteers in either case management or transmission control, and most acknowledge CDDs participation in other tasks outside control of PC-NTDs.

Training and management for CDDs was one of the most frequently addressed topics, particularly in global policy. Across the documents there was more detail on supervisory and management tasks compared to training. The more comprehensive of the WHO documents were the three training courses/modules [33,52,53]. Between them, aspects of training, MDA commodities, examples of supervision tasks, forms and recommended management styles were presented, but there was little in the way of practical guidance on how CDDs should conduct drug distribution and ancillary tasks (such as IEC) in integrated control programmes, let alone other service delivery. It is also not documented anywhere how many interventions CDDs can be responsible for. Training and supervision of CDDs was required by all the country master plans but reported variously. Training frequency ranged from annual in Nigeria and Cameroon to biannual in Ghana to quarterly in Liberia, and training was indicated for “integrated NTD activities” or “jointly for all the diseases” in some plans, and for specific functions such as “social mobilization” or “data collection reporting tools” in others. Details of CDD supervision and timing were less clearly formulated across the master plans.

Sustainability of community-based programmes relies on a committed, motivated and sufficiently incentivised cadre of volunteers to deliver drug packages year after year and extend health interventions to communities underserved by current health systems. If the expectations are not clear, and if no one has responsibility to supervise them, then it is likely they will not be inspired to continue their voluntary work [63]. Incentives and CDD motivation were mentioned in some global and regional policy documents, and in all the country master plans; however, there was little consensus or solutions offered. For example, the governing body of APOC recommended developing a policy on incentives for volunteers at country levels and urged governments to address the incentive issues [56,57]. This was reiterated in the lymphatic filariasis strategic plan, which stated: “…for volunteers… it will be necessary for health systems to create clear job descriptions, standardize incentives across programmes…”. However, other documents put the onus on the community to motivate volunteers [33]. The four country master plans all identified lack of motivation or high attrition of volunteers as threats or weaknesses to the NTD programme.

Poorly motivated volunteers and high attrition rates may be a consequence of the lack of clear guidance outlining the role of CDDs and how to train and supervise them, leading to uncertainty over responsibilities and how to perform tasks. This is a common challenge identified across empirical literature that explores the experiences of CDDs and evaluates their engagement with programme implementation. Improper training [64-66], insufficient supervision [67,68], confusion around responsibilities [69,70] and the limited amount of time allocated for workload [66,68,71] have all been reported in NTD programme literature. Recent studies and literature reviews have also highlighted that factors influencing CDD motivation and performance include and extend beyond monetary and material incentives [72-74], although this is not unique to contemporary NTD volunteer schemes [75]. Intrinsic motivation, gender, cost to participate and health systems and community support were all identified as recurring themes in the literature [73]. Community support and the health system in particular were linked to motivation and performance, and in one study the CDDs who engaged friend groups with MDA were among the best performing CDDs [74]. Despite this evidence, and the possibly increased workload for CDDs through strategic scaling-up of national NTD programmes, most of the plans did not adequately address how volunteers will be incentivised or motivated.

This lack of policy and planning frequently results in a lack of regulations or strict obligation to support CDD volunteerism, which may negatively impact their motivation and retention. To date, challenges to CDD performance based on health systems factors outlined above have been considered at the implementation level. However, this review highlights that there are also clear gaps in policy and implementation guidelines regarding the most effective ways to train, supervise and support CDDs to fulfil their role to the best of their ability. These gaps seem to have become greater overtime, indicating a reliance on programme implementers to carry out business as usual in relation to the now historic CDTI strategy. CDDs are facing changing contexts, such as changes from rural distribution to growing urbanisation, fragile and conflict affected states, migration and nomadic communities, which also have CDDs. The role of CDDs has clearly evolved over the past two decades, but the changing nature of their role has not been reflected in policy and programme documentation. For example, a recent study of an integrated NTD programme in Uganda questioned some key assumptions of CDDs in community-based MDA, including the perceived altruism of volunteers (13.56% were considered as altruistic) and the need to select volunteers through community-wide meetings (selection method had no association with treatment rates) [74]. The study demonstrated that volunteer characteristics and involvement of their friend groups may be a better way to select and monitor CDD performance [74]. This would suggest that NTD programmes need to work within existing health system infrastructure to strengthen the level of support provided to this critical cadre, whilst also considering how to document their role and management requirements clearly within programme guidelines. Agreeing the role of this cadre at the national level, as well as the development of clear training, supervision and management tools, could be a critical first step toward improving CDD performance in many settings.

Whilst our review of policy has focused on the African situation for CDDs in NTD programmes, the findings will likely apply to other global health programmes that rely on volunteers [76]. Given the diversity of health initiatives that rely on CHWs, who may also be unpaid volunteers not formally recognised by or integrated into health systems, and responsible for a myriad of tasks in the community, some ambiguity is to be expected. The recent publication of WHO guidelines on health policy and system support to optimise CHW programmes [77] is a step towards more formal recognition of their contributions in communities and the challenges faced by this cadre; however, our review has identified important issues in policy setting and programme planning that are probably reflected in other volunteer schemes operating worldwide.

For this review, we attempted to identify WHO policies and plans that include CDDs as they are known by NTD programmes, which also involved searching broader CHW documents. Therefore, a key strength of this review is that it brings together all the CDD policy documents produced over the last ten years, and indicates a key documentation gap, which is important in progressing NTD programmes. A potential weakness may lie in not reviewing national programme standard operating porcedures (SOPs) and other policies that may impact on the CDDs role or management. For example, Nigeria’s Federal Ministry of Health has produced documents such as the “training guide for trainers of community implementers (CIs) on NTD control and elimination”, while in Liberia the role of CHWs and CDDs has become more formalised, where the programme is now encouraged not to use CDDs but to implement through the new CHW policy [78]. Our review has also given less attention to the role of CDDs within other NTD service delivery, such as integrated case management, as we did not find explicit evidence of their responsibilities in this beyond that which is documented in the national master plans. However, as global and regional policy and national master plans form the basis for NTD policy setting and programme implementation guidance, we feel this review highlights critical areas that need to be addressed.

## CONCLUSIONS

Global and African WHO policy documents assume the implementation of CDDs but provide little guidance for their roles and how to prepare and utilise them within integrated programmes and changing contexts.

Given the changes in diseases covered, and adding of tasks such as morbidity control, case management, and distribution of other commodities, it seems likely the ambiguity and growth of tasks will, in the absence of clear guidance, regular training and active management at both national and local level, contribute to these programmes failing to assure equitable drug delivery to people.

## Additional material

Online Supplementary Document
