# Supplementary material for: Community drug distributors for mass drug administration in neglected tropical disease programmes: systematic review and analysis of policy documents
Source: J Glob Health. 2019 Oct 30;9(2):020414. doi: 10.7189/jogh.09.020414 (PMC6790237; doi:10.7189/jogh.09.020414)
Supplement: Online Supplementary Document [file jogh-09-020414-s001.pdf]

Supplementary material

Table S1. List of search terms and subjects used for searching World Health Organization (WHO) collections online, and the text search terms used to screen the global and regional documents using NVivo software.

**Search terms**		**Subject terms**	**NVivo text search**
**WHO.int (global)**	**WHO-AFRO library**	**WHO IRIS AFRO collections**	**Global and regional documents**
Onchocerciasis		Onchocerciasis	Distributor
River blindness		Elephantiasis, Filarial	CDD
Lymphatic filariasis		Neglected Diseases	CDDs
Elephantiasis		Schistosomiasis	Volunteer
Schistosomiasis		Intestinal, parasitic	Directed
Bilharzia		Helminth	Worker
Trachoma		Trachoma	Animator
Trichiasis		Preventive Medicine	Assistant
Soil-transmitted helminth		Preventive Health Services	Advisor
Community drug distributor		Communicable Disease Control	Implementer
CDD		Drug therapy	CMD
Community health volunteer		Delivery of Health Care	CMDs
Community health worker		Community Health Services	CHW
Community directed		Community Networks	CHWs
Preventive chemotherapy		Health Manpower	Community
Mass drug administration		Community Health Worker	CDI
Neglected tropical disease			CDTI

Table S2. Global documents supporting evidence.

Year (ref)	Type of Document[*]	Disease(s) covered	Specifying aspects of the CDDs role?	Recommendations for the CDDs role?	Strategies for training and managing CDDs?	Other comments about CDDs[†]
2010 (1)	"Strategic plan"	LF	No	None	No	Included[1]
2012 (2)	"Provisional strategy"	LF[‡]	No	An option with caveats[2]	Notes need[3]	None
2013 (3)	"Aide-memoire"	LF	No	None	Notes need[4]	None
2016 (4)	"Meeting report"	LF	No	None	Comment on aspects[5]	None
2016b (5)	"Guide"	PC-NTDs	No	None	Comment on aspects[6]	Included[7]
2013b (6)	"Handbook"	LF	No	An option[8]	Notes need	None
2010 (7)	"Manual"	PC-NTDs	Data collection[10]	None	Comment on aspects[11]	Included[12]
2011 (8)	"Manual"	LF	Data collection[13]	None	Comment on aspects[14]	Included[15]
2011 (9)	"Practical advice"	PC-NTDs	Clinical care[16]	None	No	Included[17]
2016c (10)	"Tool"	PC-NTDs	No	None	Comment on aspects[18]	Included[19]
2015 (11)	"Training course"	PC-NTDs[§]	Drug distribution; data collection; health promotion; clinical care[20]	None	Comment on aspects[21]	Included[22]
2015b (12)	"Training course"	PC-NTDs	Drug distribution; data collection; health promotion; clinical care[23]	None	Comment on aspects[24]	Included[25]

Footnote	Supporting evidence
1	"community volunteers… deliver essential medicines to many of the world's poorest people, many of whom would not be reached without volunteers. Increasing demands are being placed on volunteers as other programmes recognize their potential." (p. 41)

	"…volunteers to deliver chemotherapy for a variety of NTDs and provide other health services, it will be necessary for health systems to create clear job descriptions, standardize incentives across programmes, and coordinate efforts to build capacity". (p. 42)
2	"…lymphatic filariasis programmes could usefully contribute… community drug distributors to help improve usage and maintenance of bednets." Notes a challenge remains in areas with *Loa loa* where the CDD network still needs to be developed. (p. 9)
3	"Adequate training of… community drug distributors on mass drug administration and training on monitoring and evaluation is critical to successful implementation and evaluation" (p. 9)
4	"Planning advocacy and social mobilization activities for managing morbidity and preventing [LF] disability…": Method for community health workers and volunteers is "training sessions", the expected outcome is "awareness, commitment". (p. 24)<br>Required competences of community-based workers and volunteers are "Techniques, health promotion", and "Capacity to be built" for human resources is "Training, supervision of training facilities". (p. 25)
5	"Checklist to identify possible reasons for failure [of TAS1] and corrective measures to improve future TAS outcomes": Includes questions about drug distributor training and motivation and drug distributor:supervisor ratio; and follow up actions such as retraining drug distributors, increasing supervision and/or the ratio of supervisors:drug distributors, and collecting information on drug distributor motivation and how they are being trained. (p. 21). "…programmes can interview drug distributors… to determine whether best practices were followed during MDA (for example, was treatment directly observed? Were drug distribution platforms appropriate? Were IEC materials effective and supervision sufficient?)." (p. 7)
6	Lists example findings, potential causes, and corrective actions for interpreting and following up reported and survey coverage results. Includes causes relating to drug distributor performance, and corrective actions such as training and supervision. (pp. 37-39)
7	Glossary definition: "Reported (aka Administrative) Coverage – The coverage calculated from data reported by all drug distributors, with census figures or drug distributors reports used to estimate the population denominator." (p. 7)<br>"… -investigating additional issues (e.g.,… performance of CDDs) can generate valuable information for improving programme performance." (p. 10) "Low programme reach could be an indicator of… community drug distributor challenges…" (pp. 35-36)
8	"a community-based infrastructure of drug distributors in the lymphatic filariasis programme may be an asset for the malaria programme, as they could help to enhance net use and maintenance." (p. 61; p. 63). "Example of a tailor-made vector control plan… Long-lasting insecticidal nets programme integration with community-based drug distributors".
9	For CHWs and volunteers in LF vector control, document specifies a desired situation, "Involvement in surveys to increase use of long-lasting insecticidal nets" and a required competency, "Monitoring of bednet use; health promotion". "Steps to be taken" and "Capacity building" includes training, supervision, and M&E. (pp. 59-61)
10	"…volunteers responsible for completing the [PC tally sheet] forms must keep them safe until they are collected...". An example tally sheet for use specifically by CDDs is provided. (p. 7 and p. 15)
11	"…community volunteers who capture data… where PC is delivered deserve to be told… the results of their work." Lists reasons why this feedback is important, including "to encourage worker and community compliance for the next round of PC". (p. 11)
12	Provides examples of data quality concerns and the possible causes, including data quality concerns that relate to drug distributor recording and reporting. (p. 10)
13	"Normally, at the time of administration, drug distributors will record in their registers:", lists information recorded in the registers. "... data are compiled by the drug distributor for the village or urban area…". (p. 14)
14	"Drug distributors need to be trained and supervised to ensure that they use directly observed treatment… to maximize programme impact and to ensure that reported coverage reflects… who actually ingested the medicines." (p. 14)

	For interpreting and following up reported and surveyed coverage results, examples of findings or observations and the corrective action is indicated. Includes actions to take when CDDs may or may not be performing adequately. (p. 17)
15	Provides examples of why reported drug coverage may not reflect the actual drug coverage, which include errors in drug distributor reporting. "… the data reported from all the drug distributors are compiled and termed the reported coverage." (p. 15)
16	"simple guidance…[to] drug distributors to assist them treating patients experiencing adverse events at the drug distribution level or referring them to… health care facility." (pp. 11-12). Lists adverse events or symptoms, frequency and the treatment. (p. 37)
17	"Preparation of a large-scale preventive chemotherapy intervention includes explaining objectives and expected benefits of the intervention to... community volunteers for them to adequately inform and motivate the community and, especially, motivate all at-risk individuals to accept treatment." (p. 11)
18	"The results of the SCT should be used to develop action plans to improve the MDA, which may include… better delineation of service delivery areas for CDDs… and training for new and existing CDDs." (p. 6)<br>Provides decision rules for coverage conclusion (such as good or inadequate), interpretation and suggested next steps. Indicates drug distributor performance as a potential reason for poor coverage and how to check this. (pp. 20-21)
19	Glossary definitions: "Community drug distributors – Volunteers frequently utilized by NTD programmes to deliver preventive chemotherapy to the individuals in their community as a part of mass drug administration."<br>"Reported (aka Administrative) Coverage – The coverage calculated from data reported by all drug distributors, with census figures or drug distributors reports used to estimate the population denominator." (p. 5)<br>Provides options for SCT implementation timing and advantages and disadvantages. A disadvantage of SCT implementation immediately following MDA: "difficult to remobilize CDDs and allocate supply of drugs once MDA is believed to be finished." (p. 8)
20	At the Point of Distribution: "CDDs must give the right information; CDDs must do the right thing so as to ↓ the chances of AE/SAEs; CDDs should be able to recognize and give appropriate initial management/referral for SAEs." (Session 5.1, p. 36)<br>"CDDs play a critical role in distribution and reporting." (Session 5.1, p. 36) "Mother/CDD/HW and child should be relaxed, calm, friendly." (Session 5.2, p. 38)<br>"CDD and HWs need to be aware of issues related to administering medicines to children." and "PC Safety: Exclusion criteria MUST be understood by CDDs, HWs, and communities (social mobilization, IEC, health education). (Session 5.2, pp. 39-40)<br>"Awareness of exclusion criteria by CDDs… is essential for AE/SAE prevention and management." (Session 5.2, p. 45). "Communication Strategy: Community meetings lead by HWs/CDDs." (Session 5.3, p. 10)
21	"It is important to discuss with the implementers on the main problems: drug administration, remuneration, compliance, IEC, form filling, use of remaining drugs…". (Session 4.2, p. 14). "Effective CDD training, job aids, support, and supervision should be provided." (Session 5.1, p. 36). "Cascade training is… used when a large number of individuals (e.g. drug distributors) should be trained on a simple task." "…health workers… train the drug distributors." (Session 4.5, p. 15)<br>Lists content to include in drug distributor training: "Diseases: Very brief description of diseases targeted, MDA strategy; Drugs: What drugs to use, Dosage, including use of dose poles, How to administer, Exclusion criteria, AEs/SAEs identification, management and referral; Register/Tally Sheets" (Session 4.5, p. 11). Specifies training of CHWs is needed for community based care in lymphoedema management. (Session 2.3, p. 17)
22	Existing platforms for NTD (Oncho, LF, FBT, Trachoma) activities include: "Village Health Volunteers (VHV) distributing drug (1) at central place in the village (2) door to door" (Session 4.2, p. 4)

	"The approach of CDDs/HWs appears to be the most important determinant affecting the number of children having [AE] problems." (Session 5.2, p. 37). "Barriers to Change/Compliance… Attitude of distributors". (Session 4.4, p. 8)<br>"Benefits of integration 2: Improved supervision of community drug distributors (CDDs)… with fewer resources." (Session 4.1, p. 15). "Communities Before Round 1 of MDA: Requires special attention due to… inexperienced CDDS..." (Session 5.3, p. 13)<br>Includes examples of operational errors and lists potential causes, including "Untrained/poorly trained CDD/HW" (Session 5.3, p. 26)
23	"Drugs will be distributed by trained community distributors identified by district level personnel" (Session 7.1_7.2_7.3). "Responsibilities: CDDs – Sensitization and mobilization, Census, drug distribution" (Session 2.1_2.2). "[Who should lead this operation] Census: CDD /Community health volunteers, District health team… ; Distribution of medicines: CDD /Community health volunteers /teachers" (Session 5.1). Includes (dead) link to "Census/Medicine distribution form" – Example from Tanzania (Community Distributor's Version) (Session 5.2). "[Monitoring inventory of logistics] Who should do this?: CDD, CHW, district team" (Session 5.3). "Distribution of medicines and IEC materials: – The CDDs/teachers/health workers conduct IEC activities before and while distributing PC medicines and other service delivery (MMDP); 2. Monitoring side effects/SAE: – The CDDs/teachers/health workers record, refer to health facility and report SAE cases." (Session 5.4). "[Absentee and post-MDA follow up] Who should lead on this operation?: CDD/CHV/CHW" (Session 5.4). Provides actions for CDDs to take when AEs are encountered: "In case of adverse event(minor-moderate-severe), counsel the affected individual; Advice appropriate immediate steps and refer to nearest health center; In case of severe adverse event, urgently inform the nurse and refer the patient to the referral hospital; Plan daily visit to community after MDA for about a week daily if possible to suspect early adverse events" (Session 5.5.2). "Lymphoedema case finding: Disseminate message through... trained community health workers/volunteers going door-to-door…" (Session 6.2.1). "Hydrocele active case finding: How is it [message] disseminated? … Community health workers/volunteers going door-to-door…" (Session 6.3).
24	Includes "Steps for drug distribution by CDDs", outlining: How CDDs are recruited and selected, number of CDDs (two) per hamlet to train, and the gender ratio of CDD teams, "As much as possible choose a team of two people, a man and a woman". Includes why training CDDs is important, length of training period "between 2 days", and lists tasks the distribution teams should be trained to perform. (Session 7.1_7.2_7.3) "…training for CDDs/HWs regarding AE and SAE identification and their management" (Session 2.3). "Regular supervision and monitoring in the community… while filling out registers, collection of data from the CDDs' registers." (Session 5.4). "Health workers and community volunteers need to be given feedback a soon as possible about the results", lists reasons why this feedback is important (Session 10.5.1). Outlines the community drug distribution personnel and responsibilities, such as Field Supervisor: "Monitors the activities of CDD teams; Ensure that the distributors have the right documents (census), materials and drugs before they leave the central distribution point; Ensures that the CDD teams are working in their respective communities as per schedule" (Session 7.1_7.2_7.3). Provides examples of training CDDs need before MDA: "… recognize and respond to both AEs and SAEs; For expected AEs: inform patients that the event is almost certainly not a reaction to the medicine itself…" (Session 5.5.1).
25	"Census is undertaken to determine community populations when community distributors/ community health volunteers visit all the households in a community in order to count and register the people eligible to receive MDA (or everyone in the household)" (Session 5.2)<br>"Benefits of Integration (2): 14. Improved supervision of community drug distributors (CDDs)… with fewer resources" (Session 2.3). "Barriers: [Lists several barriers] - Attitude of distributors" (Session 4.2) "Census type: Community census data; Advantage: - More accurate if CDDs are well trained; Disadvantage: - Not always updated by CDDs" (Session 5.2). "Pre-MDA preparations in the community/School: Selection of Drug Distributors; Selected by the community members; Should be able to read and write; Resident in the community" and "Procurement and distribution of medicines and commodities to the CDDs". Provides list of MDA commodities, including: Household Registers, Posters, IEC-Kits, Flyers etc, Dose poles, drinking water and cups, Morbidity control manuals/medicines/supplies (Session 5.4). Gives examples of data quality concerns, including examples of inaccurate medicine distributor reporting (Session 10.5.1).

CDD, community drug distributor; LF, lymphatic filariasis; PC-NTDs, preventive chemotherapy neglected tropical diseases; ref, reference.

*Type of document is the words in the title that best classify the document.

†Comments about CDDs provided in the documents “background/introduction”, or in a “progress report”, an “update” or a “current situation”, were not included as other comments about CDDs in policy.

‡Loiasis and malaria are also included in document.

§Also mentions foodborne trematodiases.

Table S3. Regional documents supporting evidence.

Year (ref)	Type of Document*	Disease(s) covered	Specifying aspects of the CDDs role?	Recommendations for the CDDs role?	Strategies for training and managing CDDs?	Other comments about CDDs†
2014 (13)	"Strategy"	NTDs	No	None	No	Included[1]
2013 (14)	"Strategic plan of action"	LF and Onchocerciasis	No	None	Comment on aspects[2]	Included[3]
2007 (15)	"Meeting report"	Onchocerciasis	No	Formal recommendation[4]	No	None
2008 (16)	"Meeting report"	Onchocerciasis	No	None	No	Included[5]
2009 (17)	"Meeting report"	Onchocerciasis	No	None	No	Included[6]
2012 (18)	"Meeting report"	Onchocerciasis	No	None	Notes need[7]	Included[8]
2017 (19)	"Meeting report"	NTDs‡	No	An option[9]	No	None
2012b (20)	"Handbook"	Onchocerciasis§	Drug distribution; data collection; health promotion[10]	An option[11]	Clearly and comprehensively outlined[12]	Included[13]

Footnote	Supporting evidence
1	"Cross-cutting interventions or activities such as training, supervision, supply of medicines, IEC and sensitization campaigns, involvement of community volunteers or medicine distributors, etc., should be harmonized and streamlined to increase efficiency and avoid fragmentation." (p. 6)
2	"Key Risk [for PENDA]: CDD motivation to work on a voluntary basis is difficult to maintain over the extended period".<br>"Ways to mitigate the risk: Countries address this risk as they think fit. Some by offering incentives, some by seeking synergies with other programmes that offer incentives to create a common platform for CDD engagement." (p. 38)
3	"Objective 5: Reduce suffering and disability through morbidity management and disability prevention<br>Activity area 5.1: Establish or strengthen community structures to support morbidity management<br>The involvement and collaboration of patients and their families, community volunteers and community health workers is essential." (p. 31)
4	"The JAF strongly endorsed an increase in the recommended ratio of 1 CDD to treat a maximum of 100 persons from the current ratio of 1 CDD to treat a maximum of 250 persons." (p. 7)
5	"To address the re-occurring issue of incentives for CDTI volunteers, JAF recommended the development of a policy on incentive for volunteers at country levels, in order to eliminate the risk of negative competition among programmes." (p. 3)

6	"Decision: JAF thanked the NGDO Group for their continued support to the communities and countries and urged Governments to address the issues related to incentives for CDDs." (p. 6)
7	"… dedicate more resources to ensure that more women receive training in community directed treatment with ivermectin and are community directed distributors as well… the next [APOC] report should show a significant increase in this regard…" (p. 4)
8	"…for eye care… CDDs are often utilised in case finding." (p. 8)
9	Action Points and recommendations: "Conduct annual joint planning and identify opportunities for integration of field interventions… (e.g.,… Sensitization (IEC) on skin NTDs during Mass Medicine Administration (MMA) campaigns by community distributors.)" (p. 3)
10	"The community service provider has a responsibility to the community that selects him/her to perform specific tasks: a) attend training and other educational programmes in respect of the intervention programme; b) provide information, education and communication to the community; c) conduct census and estimate the population that will need the service; d) collect or ensure that intervention commodities are available in the community; e) keep record of how the intervention was delivered; f) submit a record of intervention to the supply collection point; g) provide feedback to the community" (p. 73).<br>"Implementation of the intervention by community implementers: a) census taking for information on quantity of intervention materials required; b) collection of intervention materials; c) delivery of the interventions; d) record keeping." (p. 35).<br>"Chief among the roles of the communities is to select members of the community to be trained by the health workers on the delivery of specific health interventions. Those community members so trained have the task of ensuring that everyone eligible for the intervention commodity does not only access the intervention in good quantity and quality but also adhere to the recommended use of the commodities" (p. 54). "One of the first tasks performed after CDDs are trained is their mobilization of the community to conduct a community census and develop that into a community register." (p. 91). "All CDD will provide/monthly/quarterly activity reports to district level" (p. 147). Provides an example of a "Tally Sheet for Literate Volunteers" and "CDTI Tally Sheet for Low Literacy CDDs" (p. 152). "…the community service provider (also referred to as community resource person, CDD or by other names)" (p. 70). See also p. 54.
11	Some options were provided throughout the document, for example: "Since the community is in charge of decision making, the community decides how tasks get done. This may mean that several people are engaged as volun-teers. Maybe one older woman volunteers to provide intermittent preventive therapy (IPTs) to pregnant women and counsel them on safe pregnancy. Maybe a man with a motorcycle volunteers to maintain LLIN supplies and carry out their distribution. A mother in the community might volunteer to do the community case management of malaria, pneumonia and diarrhoea because she has a good way of handling small children (p. 96).
12	See units six, seven and eight for "Setting up the CDI strategy", "Supervision" and "Monitoring and Evaluation", respectively.<br><br><u>Training:</u> For "training community selected members for implementing CDI", the document states "Refer to the existing APOC training manual for CDDs", with use of audio visuals by the GAELF and SCI where the training covers multiple health interventions (p. 118). Provides list of other audio-visuals, "Use of Training video of APOC Training community-directed distributors (CDDs) of ivermectin", "Training videos on LF, Trachoma and Schistosomiasis…", and other training manuals (pp. 118-120). "CDD training should include… how to keep record and submit monthly summary forms." and "… a component of supply management." (pp. 90-91). Specifies the number of days that should be allocated for training CDDs, including one day of practical (p. 118).<br><br><u>Management:</u> "The role of a supervisor" (pp. 132-133) and different supervision styles: "Coaching supervision involves high directive and high support behaviour… this is suitable to use in the CDI process when dealing with community implementers." (p. 131). States the supervisor role:

	“Frontline Health Facility – Supervise community distributors twice monthly; check CDD record”, with a list of tools required for supervisors (p. 177) and forms, including an ‘Integrated supervision checklist for PHC programmes’, ‘Supervision checklist for CDTI’, ‘Monitoring checklist for CDTI’, ‘Monitoring checklist for CDTI’, ‘Community summary form’, ‘Summary of sub-district/FLHF activities’, ‘Community self-reporting form’ (pp. 183-193).<br>Provides examples of types of problems that can be identified during supervision and possible solutions/actions (p. 180), and examples of supervision tasks for CDD distribution (p. 181). “One method for regular data collection is holding of a monthly CDD review and supervisory meetings where CDDs bring their summary forms.” (p. 90).
13	General statements on CDDs or community implementers were provided throughout the document, such as:<br>CDD motivation and incentives (p. 55);<br>CDD selection, “At this meeting the CDI process is explained to the community members including resource mobiliza-tion, selection criteria for the community representatives/volunteers,” (p. 96) and “Emphasize- that it is the responsibility of the community to decide who should be a distributor.” (p.117);<br>Mode of drug distribution: “1. The community may choose that their volunteers go from door-to-door to deliver services to those at need; 2. Community members visit the volunteers at home to obtain the available service; and finally 3. The community can choose the option of meeting the volunteers at the community square/hall at agreed time and day to receive services. Whatever option the community decides to adopt has implication in the kind of support that the volunteers would need. Example, if the community decides for the first option, the CDDs might need transportation support to visit households to deliver services, etc.” (pp. 94-95).<br>Gender mainstreaming.

CDD, community drug distributor; LF, lymphatic filariasis; NTD, neglected tropical diseases; ref, reference.

*Type of document is the words in the title that best classify the document.

†Comments about CDDs provided in the document “background/introduction”, or in a “progress report”, an “update” or a “current situation”, were not included as other comments about CDDs in policy.

‡Addresses preventive chemotherapy-NTDs and case management-NTDs.

§Also mentions other diseases that may use community-directed interventions, such as lymphatic filariasis, schistosomiasis, trachoma and malaria (bed nets).

References

1. World Health Organization. Lymphatic filariasis: progress report 2000–2009 and strategic plan 2010–2020. Geneva: World Health Organization; 2010. Available: http://www.who.int/lymphatic_filariasis/resources/9789241500722/en/.
2. World Health Organization. Provisional strategy for interrupting lymphatic filariasis transmission in loiasis-endemic countries: report of the meeting lymphatic filariasis, malaria and integrated vector management, Accra, Ghana, 5-9 March 2012. Geneva: World Health Organization; 2012. Available: http://www.who.int/lymphatic_filariasis/resources/who_htm_ntd_pct_2012.6/en/.
3. World Health Organization. Lymphatic filariasis: managing morbidity and preventing disability – An aide-memoire for national programme managers. Geneva: World Health Organization; 2013. Available: http://www.who.int/lymphatic_filariasis/resources/9789241505291/en/.
4. World Health Organization. Responding to failed transmission assessment surveys. Report of an ad hoc meeting: strategic and technical advisory group for neglected tropical diseases subgroup on disease-specific indicators, 4 December 2015, Washington (DC), USA. Geneva: World Health Organization; 2016. Available: http://www.who.int/lymphatic_filariasis/resources/9789241511292/en/.
5. World Health Organization. Coverage Evaluation surveys for preventive chemotherapy: field guide for implementation. Geneva: World Health Organization; 2016. Available: https://www.ntdsupport.org/resources/coverage-survey-builder-coverage-evaluations.
6. World Health Organization. Lymphatic filariasis: Practical entomology – A handbook for national elimination programmes. Geneva: World Health Organization; 2013. Available: http://www.who.int/lymphatic_filariasis/resources/9789241505642/en/.
7. World Health Organization. Monitoring drug coverage for preventive chemotherapy. Geneva: World Health Organization; 2010. Available: http://www.who.int/neglected_diseases/preventive_chemotherapy/monitoring_evaluation_manual/en/.
8. World Health Organization. Lymphatic filariasis: monitoring and epidemiological assessment of mass drug administration - A manual for national elimination programmes. Geneva: World Health Organization; 2011. Available: http://www.who.int/lymphatic_filariasis/resources/9789241501484/en/.
9. World Health Organization. Assuring safety of preventive chemotherapy interventions for the control of neglected tropical diseases: practical advice for national programme managers on the prevention, detection and management of serious adverse events. Geneva: World Health Organization; 2011. Available: http://www.who.int/neglected_diseases/resources/9789241502191/en/.
10. World Health Organization. Rapid monitoring of preventive chemotherapy coverage in neglected tropical disease programmes: supervisor's coverage tool. Geneva: World Health Organization; 2016. Available: https://www.ntdsupport.org/resources/supervisors-coverage-tool.

11. World Health Organization. Programme managers' training course for NTDs targeted for control or elimination by preventive chemotherapy interventions. Available from: http://www.who.int/neglected_diseases/training/programme_managers/en/.
12. World Health Organization. District level management NTD training course. Available: http://www.who.int/neglected_diseases/training/modules_for_district_level_management/en/.
13. World Health Organization Regional Office for Africa. Regional strategy on neglected tropical diseases in the WHO African Region 2014-2020. Brazzaville: World Health Organization Regional Office for Africa; 2014. Available: https://www.afro.who.int/publications/regional-strategy-neglected-tropical-diseases-who-african-region-2014-2020.
14. African Programme for Onchocerciasis Control-World Health Organization. Programme for the Elimination Neglected Diseases in Africa (PENDA): strategic plan of action and indicative budget 2016-2025. Ouagadougou: African Programme for Onchocerciasis Control/World Health Organization; 2013. Available: http://www.who.int/apoc/en_apoc_strategic_plan_2013_ok.pdf.
15. African Programme for Onchocerciasis Control. Thirteenth session of the Joint Action Forum, Brussels, Belgium, 4-7 December 2007 - Final communique. Available: http://www.who.int/apoc/about/structure/jaf/en/.
16. African Programme for Onchocerciasis Control. Fourteenth session of the Joint Action Forum, Kampala, Uganda, 8-11 December 2008 – Final communique. Available: http://www.who.int/apoc/about/structure/jaf/en/.
17. African Programme for Onchocerciasis Control. Fifteenth session of the Joint Action Forum, Tunis, Tunisia, 8-10 December 2009 - Final communique. Available: http://www.who.int/apoc/about/structure/jaf/en/.
18. African Programme for Onchocerciasis Control. Eighteenth session of the Joint Action Forum, Bujumbura, Burundi, 11-13 December 2012 – Final communique. Available: http://www.who.int/apoc/about/structure/jaf/en/.
19. World Health Organization Regional Office for Africa. Summary report, action points and recommendations of the first joint meeting on preventive chemotherapy and case management NTDs, Libreville, Gabon, 20-22 June 2017. Available: http://espen.afro.who.int/system/files/content/resources/Summary_And_Recommendations_of_JointPC_AndCM_NTD_Prog_Mgrs_Meeting_June2017_English.pdf.
20. African Programme for Onchocerciasis Control. Curriculum and training module on the community-directed intervention (CDI) strategy for faculties of medicine and health sciences - Trainers' handbook. Ouagadougou: African Programme for Onchocerciasis Control/World Health Organization; 2012. Available: http://www.who.int/apoc/publications/APOC_Handbook_CDI_EN/en/.
